# High contrast sensitivity for visually guided flight control in bumblebees

**DOI:** 10.1007/s00359-017-1212-6

**Published:** 2017-09-06

**Authors:** Aravin Chakravarthi, Almut Kelber, Emily Baird, Marie Dacke

**Affiliations:** Department of Biology, Lund University, Sölvegatan 35, Lund, Sweden

**Keywords:** *Bombus terrestris*, Hymenoptera, Spatial resolution, Contrast sensitivity, Motion detection system

## Abstract

Many insects rely on vision to find food, to return to their nest and to carefully control their flight between these two locations. The amount of information available to support these tasks is, in part, dictated by the spatial resolution and contrast sensitivity of their visual systems. Here, we investigate the absolute limits of these visual properties for visually guided position and speed control in *Bombus terrestris*. Our results indicate that the limit of spatial vision in the translational motion detection system of *B. terrestris* lies at 0.21 cycles deg^−1^ with a peak contrast sensitivity of at least 33. In the perspective of earlier findings, these results indicate that bumblebees have higher contrast sensitivity in the motion detection system underlying position control than in their object discrimination system. This suggests that bumblebees, and most likely also other insects, have different visual thresholds depending on the behavioral context.

**Electronic supplementary material:**

The online version of this article (doi:10.1007/s00359-017-1212-6) contains supplementary material, which is available to authorized users.

## Introduction

Vision guides a broad range of behavior in flying insects, from locating food sources and potential mates to navigation and flight control. Two fundamental and interrelated aspects of spatial vision are spatial resolution (the ability to distinguish two closely spaced stimuli) and contrast sensitivity (the ability to detect the minimum contrast between the stimuli and their background) (De Valois and De Valois 1990). The nature of the relationship between spatial resolution and contrast sensitivity sets limits for visual systems that must strive to optimize the acquisition of the information necessary to guide behavior. For example, at the spatial resolution limit, only high contrasts can be detected. To fully understand the visual world of insects it is therefore important to investigate not only the limits of their spatial resolution, but also their contrast sensitivity at different spatial frequencies.

Not all visually guided behaviors use the highest possible resolution, and different behavioral tasks may indeed extract different information from the visual system. For example, from anatomical measures (Somanathan et al. 2009), the resolution of the Asian honeybee *Apis cerana* can be estimated as about 0.8 cycles deg^−1^, while behavioral investigations have determined the point object resolution to be lower at 0.26–0.36 cycles deg^−1^ (Zhang et al. 2014). Task-dependent variations may also occur in contrast sensitivity, as evidenced by studies in humans (Robson 1966; Kelly 1979; Barten 1993) and birds (Haller et al. 2014).

Behavioral investigations of the visual abilities of bees have typically focused on single object resolution using dual choice discrimination tasks (Spatial resolution: Giurfa et al. 1996; Spaethe and Chittka 2003; Dyer et al. 2008; Wertlen et al. 2008; Zhang et al. 2014; Spatial resolution and contrast sensitivity: Srinivasan and Lehrer 1988; Macuda et al. 2001; Chakravarthi et al. 2016). However, little is known about the spatial resolution and contrast sensitivity limits of other visually guided behaviors, such as flight control. In tethered flying *Drosophila*, the spatial, temporal and contrast sensitivity of the turning optomotor response varies depending on the type of motion being presented (Duistermars et al. 2007), but it is not clear how this relates to the regulation of other behaviors such as speed and position control.

In a dual choice discrimination paradigm, the buff-tailed bumblebee *Bombus terrestris* had a peak contrast sensitivity (defined as the inverse of contrast threshold, i.e., minimal noticeable contrast) of around 1.57 for sinusoidal gratings of 0.09 cycles deg^−1^ spatial frequency (Chakravarthi et al. 2016). This remarkably low contrast sensitivity is surprising because, although this might be sufficient for detecting flowers to feed from, it would be far less suitable for detecting the pattern of visual motion, or translational optic flow (TOF) that flying bumblebees rely on to control their ground speed and position (Baird et al. 2010; Dyhr and Higgins 2010; Baird and Dacke 2012; Linander et al. 2015, 2016). This is because TOF is optimally sampled with low resolution and high contrast sensitivity (Srinivasan and Bernard 1975). One potential explanation for this paradox is that spatial resolution and contrast sensitivity in bumblebees might vary depending upon the behavioral task. Evidence for such context-dependent differences in contrast sensitivity has been provided by studies in birds (Haller et al. 2014) and humans (Robson 1966; Kelly 1979; Barten 1993). Whether insects also have the ability to adapt their contrast sensitivity to the behavioral task remains unknown.

The aim of the present study is to determine the limits of spatial resolution and contrast sensitivity in the flight control behavior of *Bombus terrestris* and to compare this to the findings of Chakravarthi et al. (2016) that used similar patterns to determine the same parameters for an object detection task. A well-established method to study flight control in bees is by analyzing how they control their lateral position as they fly along tunnels that display visual textures on each wall (Srinivasan et al. 1991). In such tunnels, bumblebees regulate their lateral position—and thus their distance to the tunnel walls—by balancing the magnitude of TOF experienced in each eye (Dyhr and Higgins 2010). Here, we investigate the effect of changes in spatial frequency and contrast on the lateral position of bumblebees by keeping one wall of a flight tunnel featureless gray, while varying the visual features displayed on the other wall. We find that, when both walls are gray, or when the bees cannot perceive the texture on the other wall (when the spatial frequency is too fine or the contrast is too low), the flight trajectories are clustered about the tunnel’s midline. When the bees can perceive the texture on the test pattern, they fly closer to the gray wall in an attempt to balance the relative TOF experienced in each eye. Overall, we find that the spatial resolution limit for lateral position control is similar to that recorded for object detection but that contrast sensitivity is drastically higher, suggesting that the visual limits of bumblebees do indeed change depending on the behavioral task.

## Materials and methods

### Animals and experimental setup

Experiments were performed using medium-sized bumblebees (see below) from five colonies of *Bombus terrestris* (Koppert, UK). The hive was placed indoors in an aluminum net cage (2.3 m wide, 2.0 m high and 2.0 m deep; 600 lx, 20 ℃, 10:14 L:D cycle). Bees were trained to fly along an experimental tunnel (200 cm long, 30 cm wide, 30 cm high) to a sucrose feeder (5 cm wide, 28 cm long, 4 cm deep) that was placed at the end in a white plastic box such that it was not visible to the bees while flying towards it. The bees were allowed to feed from the sucrose (30% concentration) ad libitum, after which they returned to the hive. The top of the tunnel was covered with netting and the floor was lined with white matte laminated paper.

### Experimental procedure

During each experimental trial, one wall displayed a uniform 40% gray pattern (‘constant wall’) while the other wall displayed the test pattern (‘variable wall’). The test patterns were presented in a pseudo-randomized order and the side (left or right) of the ‘variable wall’ was alternated to minimize the risk of side bias. To investigate the spatial resolution of the visual flight control system of bumblebees, the ‘variable wall’ was either uniform gray or displayed a high contrast sinusoidal grating (Michelson contrast 87%; see below) of 0.10, 0.15, 0.20, 0.40, 0.65, 0.80 or 2.0 cycles cm^−1^ pattern wavelength which equals spatial frequencies of 0.03, 0.04, 0.05, 0.10, 0.17, 0.21 and 0.52 cycles deg^−1^ as measured from the midline of the tunnel. These stimuli were printed onto Marnello paper using a large format HP DesignJet Z6200 printer.

To investigate the contrast sensitivity of the visual flight control system of bumblebees, the ‘variable wall’ was either uniform gray or displayed a sinusoidal grating of 0.10, 0.15, 0.20 or 0.40 cycles cm^−1^, each printed with Michelson contrasts (see below) of 39, 22, 14, and 3%. The maximum (*I*_Max_) and minimum (*I*_Min_) intensities of the gratings were measured using a photometer (Hagner ScreenMaster, B. Hagner, Solna, Sweden) with the human photopic spectral sensitivity. The Michelson contrasts (Michelson 1927) of all gratings (MC) were calculated as:

1 $${\text{MC}} = \frac{{I_{\text{Max}} - I_{\text{Min}} }}{{I_{\text{Max }} + I_{\text{Min}} }}$$

To minimize any potential learning effects associated with the previous test condition, the bees were allowed to forage at the feeder for 30 min before each trial commenced. During this time both walls were covered with randomized black-and-white checkerboard patterns.

In each trial, flights to the feeder were filmed at 50 Hz using a camera (Sony HDR-CX730) mounted 150 cm above the tunnel. To minimize the risk of pseudo-replication, we recorded 25–30 flights in each condition, which typically took 15–30 min. As a foraging bout typically took about 4–5 min for each bee (as measured during this experiment) and we normally analyzed flights from at least six different bees during this time window, we estimate that these 25–30 flights were performed by an absolute minimum of six different individuals. However, as there were many different foragers flying to the feeder during the experiments, the likelihood of including many flights from the same individuals is minimal. In addition, previous studies with marked bumblebees have shown that intra-individual variation between flights is not different from inter-individual variation (e.g., Dyhr and Higgins 2010).

### Analysis

The bee’s position in each frame was recorded over 100 cm spanning the tunnel’s mid-section using a tracking program (Lindemann 2005). Pixel coordinates were converted to mm using a calibration factor obtained from images of a check pattern of known size placed 15 cm above the tunnel floor.

As the bees do not necessarily fly along the midline of the tunnel in all experimental conditions, we calculated the apparent spatial frequency of each sinusoidal grating, here defined as the spatial frequency experienced from the true lateral position of the bees. The true lateral position could be calculated in two ways either (1) from the median of the average lateral distances from the variable wall recorded for each flight in each condition or (2) as a median of the apparent spatial frequencies experienced for all bees for every lateral position recorded. In this study, these two sets of calculations resulted in nearly identical values (Table 1) and the median of the average distance from the variable wall was recorded for each flight was thus used as a measure for apparent spatial frequency throughout the manuscript. To account for possible asymmetries outside the flight tunnel, in the control condition (with gray pattern on both walls), the lateral distance was measured from the left wall for half of the flights, and from the right wall for the other half (flights were randomly assigned one or the other group).

**Table 1:** Apparent spatial frequencies of sinusoidal gratings

Pattern wavelength (cycles cm^−1^)	Apparent spatial frequency [median (IQR)] (cycles deg^−1^)	
	For average distance from wall^a^	For point to point distance from wall
0.10	0.04 [0.04 0.04]	0.04 [0.04 0.04]
0.15	0.06 [0.06 0.07]	0.06 [0.05 0.07]
0.20	0.07 [0.07 0.08]	0.07 [0.06 0.08]
0.40	0.13 [0.12 0.15]	0.13 [0.11 0.15]
0.65	0.21 [0.19 0.23]	0.21 [0.18 0.23]
0.8	0.22 [0.20 0.24]	0.22 [0.19 0.25]
2.0	0.57 [0.49 0.66]	0.60 [0.47 0.73]

^a^Used for further analysis of the effect of spatial frequency on lateral position and ground speed

It is important to note that the allotheism that exists among the workers of *Bombus terrestris* affects their spatial resolution (Spaethe and Chittka 2003). In this study, we therefore limited our analysis to medium-sized bees by only including flights from bees that occupied an average body width of 7–8 pixels in the recorded video. This corresponds to an inter-tegular width of about 4 mm. Asymmetrical conditions were presented twice (with the grating on the left or right wall), but as these conditions were qualitatively similar, the data from these conditions were pooled for the analysis. The symmetric control condition was presented once, but the left and right walls were each taken as ‘constant wall’ for half the flights (see above). Statistical comparisons were performed using ANOVA with Dunnett’s post hoc multiple comparison test (Quinn and Keough 2002). The independent variable was the test pattern and the dependent variable was position or ground speed.

## Results

### Effect of spatial frequencies on lateral position in *Bombus terrestris*

**Fig. 1:**
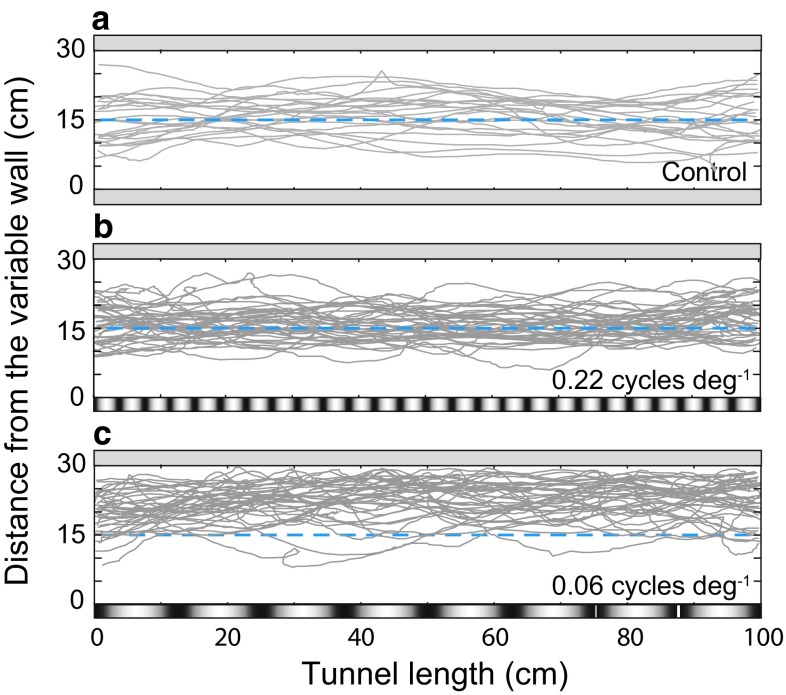
Examples of individual flight trajectories of *Bombus terrestris.* The bees flew down a tunnel with a uniform *gray* ‘constant wall’ (*top*) and a ‘variable wall’ (*bottom*) that was either **a** uniform *gray* or **b**, **c** carried a *black* and *white* sinusoidal grating of a given apparent spatial frequency [true spatial frequency]; **b** 0.22 cycle deg^−1^ [0.8 cycles cm^−1^] and **c** 0.06 cycle deg^−1^ [0.15 cycles cm^−1^]. The spatial frequency in **b** is above the spatial threshold of the bees. The *blue dashed line* indicates the midline of the tunnel and pattern frequency shown is not to scale

To analyze the effect of spatial frequency on lateral position in *B. terrestris,* we recorded flights along the tunnel when the ‘constant wall’ displayed a uniform gray pattern and the ‘variable wall’ displayed either a uniform gray pattern (control) or sinusoidal gratings of different spatial frequencies (0.10, 0.15, 0.20, 0.40, 0.65, 0.80 or 2.0 cycles cm^−1^). In the control condition, when both walls of the tunnel were uniform gray, the flights were centered on the midline (Fig. 1a, Table S1 in Supplementary information). When the variable wall displayed sinusoidal gratings of 0.8 cycles cm^−1^, the flights were still centered about the midline and were not significantly different from the control condition (Fig. 1b, ANOVA with Dunnett’s post hoc multiple comparison; *p* > 0.05, Table 2 for statistical details). This suggests that the bees cannot resolve this fine grating, and that instead, the patterned ‘variable wall’ of the tunnel appeared gray to them. However, when the spatial frequency of the gratings on the variable wall was 0.65 cycles cm^−1^, or lower, the bees flew closer to the gray ‘constant wall’ than in the control condition (Fig. 1c, ANOVA with Dunnett’s post hoc multiple comparison *p* < 0.0001, Table 2 for statistical details). This change in flight position is most likely an attempt to balance the relative magnitude of optic flow from the striped wall (strong optic flow cues) and the gray wall (weak optic flow cues).

**Table 2:** Effect of spatial frequency on lateral position and ground speed control

Lateral position [ANOVA with Dunnett’s post hoc multiple comparison with control (gray walls)]							
* F*_7,346_ = 30.54; *p* < 0.0001; *n* ^a^ = 354							
Spatial frequency (cycles deg^−1^)	0.04	0.06	0.07	0.13	0.21	0.22	0.57
Total number of flights	45	46	45	47	55	42	50
Significance level	***p*** **<** **0.0001**	***p*** **<** **0.0001**	***p*** **<** **0.0001**	***p*** **<** **0.0001**	***p*** **=** **0.003**	*p* = 0.99	*p* = 0.08
Ground speed [ANOVA with Dunnett’s post hoc multiple comparison with control (gray walls)]							
* F*_7,346_ = 7.10; *p* < 0.0001; *n* = 354							
Spatial frequency (cycles deg^−1^)	0.030	0.040	0.050	0.100	0.170	0.210	0.524
Significance level	*p* = 0.83	***p*** **<** **0.01**	***p*** **<** **0.0001**	*p* = 0.25	*p* = 0.67	*p* > 0.99	*p* = 0.91

Significant values are indicated in bold

^a^Total no. of flights (including the control data, *n* = 24) analyzed

**Fig. 2:**
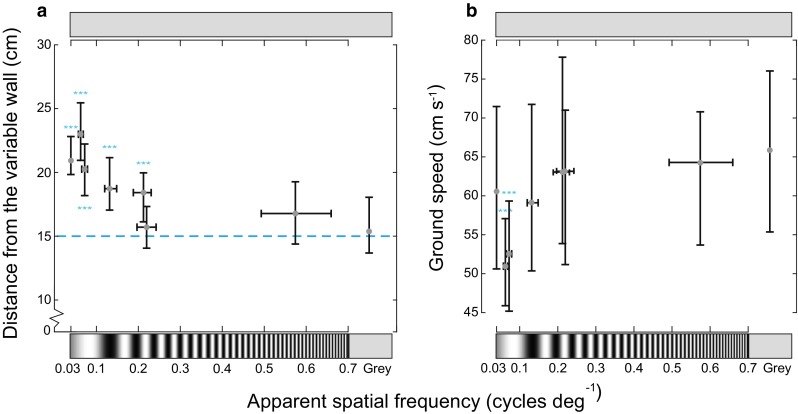
Effect of spatial frequencies on lateral position and ground speed in *Bombus terrestris*. The bees flew through a tunnel where the ‘constant wall’ (*top*) displayed a uniform *gray* pattern and the ‘variable wall’ (*bottom*) displayed a test pattern of a given spatial frequency or a uniform gray control pattern. The *blue dashed line* indicates the midline of the tunnel. Apparent spatial frequency is calculated from the median of the average lateral distances from the patterned wall recorded for each flight in each condition. **a** *Gray filled circles* show the median lateral position of the bees along the *y*-axis and the median apparent spatial frequency along the *x*-axis. Pattern frequencies shown are not to scale. Whiskers along the *y*- and *x*-axis represent second and third quartile of the average positions of the individual trajectories and the apparent spatial frequency, respectively. **b** *Gray filled circles* show the median ground speed of the bees along the *y*-axis and the median apparent spatial frequency along the *x*-axis. Whiskers along the *y*- and *x*-axis represent second and third quartile of the average ground speed of the individual trajectories and the apparent spatial frequency, respectively. *n* (total number of flights) = 354, ***p < 0.001

The apparent spatial frequencies of the different gratings, as perceived by the bees from their flight positions in the tunnels, were 0.04 [0.04 0.04], 0.06 [0.06 0.07], 0.07 [0.07 0.08], 0.13 [0.12 0.15], 0.21 [0.19 0.23], 0.22 [0.20 0.24] or 0.57 [0.49 0.66] [median (interquartile range)] cycles deg^−1^ (Table 1). The bees thus still steered away from a wall displaying a grating of an apparent spatial frequency of 0.21 cycles deg^−1^ (0.65 cycles cm^−1^) but not from a wall displaying an apparent spatial frequency of 0.22 cycles deg^−1^ (0.8 cycles cm^−1^) or 0.57 cycles deg^−1^ (2.0 cycles cm^−1^) (Fig. 2a). This suggests that the spatial resolution of the motion detection system mediating position control in *B. terrestris* lies approximately at 0.21 cycles deg^−1^.

Next, we analyzed the effect of apparent spatial frequency on ground speed, which is also controlled using TOF in bumblebees (Baird et al. 2010). However, our results suggest that unlike lateral position, ground speed did not vary consistently with spatial frequency (Fig. 2b and Table 2 for statistical details). The inconsistency of the response does not allow us to draw any conclusions about the relationship between ground speed and spatial frequency from this experiment, an observation that is consistent with the findings of similar experiments performed in *B. impatiens* (Dyhr and Higgins 2010). We therefore do not consider it useful to include analyses of ground speed in the subsequent experiment.

### Effect of spatial frequency and pattern contrast on lateral position in *B. terrestris*

**Fig. 3:**
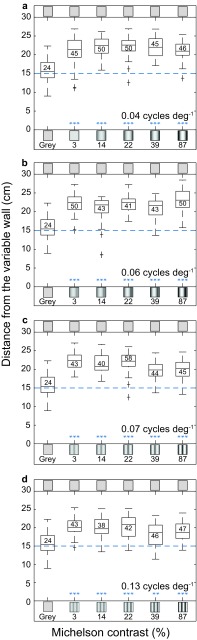
Effect of pattern contrast on lateral position in *B. terrestris*. The bees flew through a tunnel where the ‘constant wall’ (*top*) displayed a uniform gray pattern and the ‘variable wall’ (*bottom*) displayed a grating of a given Michelson contrast ranging from 0 to 87%. *Boxplots* show the distribution of lateral positions recorded for gratings of **a** with an apparent spatial frequency of 0.04 cycles deg^−1^ (0.10 cycles cm^−1^) (*n* = 260), **b** with an apparent spatial frequency of 0.06 cycles deg^−1^ (0.15 cycles cm^−1^) (*n* = 251), **c** with an apparent spatial frequency of 0.07 cycles deg^−1^ (0.20 cycles cm^−1^) (*n* = 254) and **d** with an apparent spatial frequency of 0.13 cycles deg^−1^ (0.40 cycles cm^−1^) (*n* = 240). The *number* inside each box represents the number of flights analyzed. Pattern frequency and contrast are not to scale.* Whiskers* represent second and third quartile with the median shown with a *black line* inside the box. The outliers are shown by *plus* sign. **p < 0.01; ***p < 0.001

To analyze the effect of contrast on lateral position, we recorded flights along the tunnel when the ‘constant wall’ was uniform gray and the ‘variable wall’ displayed a sinusoidal grating of a given combination of spatial frequency (0.10, 0.15, 0.20 or 0.40 cycles cm^−1^ with apparent spatial frequencies of 0.04 [0.04 0.04], 0.06 [0.06 0.07], 0.07 [0.07 0.08], 0.13 [0.12 0.15] [median (interquartile range)] cycles deg^−1^) and contrast (87, 39, 22, 14, 3%). For most combinations, the bees flew significantly closer to the gray ‘constant wall’ than in the control condition (Fig. 3, ANOVA with Dunnett’s post hoc multiple comparison, *p* < 0.001; Table 3 for statistical details; Table S2 in Supplementary information). This suggests that the motion detection system used to control position is sensitive to visual stimuli of contrasts of 3% or possibly even lower, translating into a contrast sensitivity of at least 33.

**Table 3:** Effect of contrast on lateral position

Lateral position [ANOVA with Dunnett’s post hoc multiple comparison with control (gray walls)]					
Spatial frequency: 0.04 cycles deg^−1^; *F*_5,254_ = 21.93; ***p*** **<** **0.0001**; *n* ^a^ = 260					
Contrast (%)	3	14	22	39	87
Significance level	***p*** **<** **0.0001**	***p*** **<** **0.0001**	***p*** **<** **0.0001**	***p*** **<** **0.0001**	***p*** **<** **0.0001**
Spatial frequency: 0.06 cycles deg^−1^; *F*_5,245_ = 24.38; ***p*** **<** **0.0001**; *n* ^a^ = 251					
Contrast (%)	3	14	22	39	87
Significance level	***p*** **<** **0.0001**	***p*** **<** **0.0001**	***p*** **<** **0.0001**	***p*** **<** **0.0001**	***p*** **<** **0.0001**
Spatial frequency: 0.07 cycles deg^−1^; *F*_5,248_ = 27.24; ***p*** **<** **0.0001**; *n* ^a^ = 254					
Contrast (%)	3	14	22	39	87
Significance level	***p*** **<** **0.0001**	***p*** **<** **0.0001**	***p*** **<** **0.0001**	***p*** **<** **0.0001**	***p*** **<** **0.0001**
Spatial frequency: 0.13 cycles deg^−1^; *F*_5,234_ = 11.68; ***p*** **<** **0.0001**; *n* ^a^ = 240					
Contrast (%)	3	14	22	39	87
Significance level	***p*** **<** **0.0001**	***p*** **<** **0.0001**	***p*** **<** **0.0001**	***p*** **=** **0.002**	***p*** **<** **0.0001**

Significant values are indicated in bold

^a^Total no. of flights (including the control data, *n* = 24) analyzed

## Discussion

### The spatial resolution limit is similar for position control and object discrimination

Overall, our results indicate that the lower limit for spatial resolution in the motion detection system underlying position control in *Bombus terrestris* lies somewhere around 0.21 cycles deg^−1^. This is similar to the spatial resolution recently identified for the object discrimination system in this species (≈0.21 cycles deg^−1^) (Chakravarthi et al. 2016) and is only somewhat higher than that estimated for *B. impatiens* (0.14 cycles deg^−1^) in similar types of free flight experiments (Dyhr and Higgins 2010).

It is important to note that the resolution limit of 0.21 cycles deg^−1^ [as well as the limit reported for *B. impatiens* by Dyhr and Higgins (2010)] is calculated for a 90° lateral viewing angle. However, since *B*. *terrestris* has recently been shown to utilize optic flow cues flow for position control more frontally (around 28° lateral viewing angle) (Linander et al. 2015), it is possible that the spatial resolution threshold of the motion detecting system is actually even somewhat higher. Unfortunately, until we understand more about what determines the viewing angle at which bumblebees measure TOF, it will not be possible to make a more accurate estimate of this threshold. Our measurement of around 0.2 cycles deg^−1^ spatial resolution as calculated for a 90° viewing angle, therefore, represents a conservative lower limit of the visual capabilities of bumblebees in this type of free flight experiment.

### Motion improves contrast sensitivity during position control

In the present study, the bees were able to adjust their position in relation to sinusoidal gratings down to contrasts as low as 3%. This held true for all sinusoidal gratings tested (0.10, 0.15, 0.20 or 0.40 cycles cm^−1^, with spatial frequencies between 0.04 and 0.13 cycles deg^−1^) and equals a contrast sensitivity of at least 33 across a broad range of spatial frequencies. In a similar experiment, Dyhr and Higgins (2010) found that larger individuals of the closely related bumblebee species *B. impatiens* detect and steer away from grating patterns down to a contrast of 5% (see Fig. 5 in Dyhr and Higgins 2010), equaling a contrast sensitivity of 20.

Using sinusoidal gratings similar to those used in this study (but with frequencies of 0.09, 0.13 and 0.18 cycles deg^−1^), Chakravarthi et al. (2016) found the peak contrast sensitivity for the object discrimination system in *B. terrestris* to be 1.61 (62% contrast) at 0.08 cycles deg^−1^ (Chakravarthi et al. 2016). This is almost 21 times lower than our estimate for the contrast sensitivity of the translational motion detecting system underlying position control. This suggests that visually mediated control systems of *B. terrestris* operate with different contrast thresholds that are set by task-specific neural circuits rather than by physical constraints of the compound eye.

Contrast sensitivity thresholds in insects are often measured physiologically from neurons in the brain of a stationary animal viewing wide-field rotational or translational stimuli. From these types of recordings, a contrast sensitivity threshold around 20 has been identified for the bumblebee *B. lapidarius* (O’Carroll et al. 1996). This is somewhat lower than the sensitivity measured in this study, but far higher than the peak contrast sensitivity (1.61) reported for the object discrimination system of *B. terrestris* (Chakravarthi et al. 2016). Using dual choice tests, Srinivasan and Lehrer (1988) found that the object detection system of the European honeybee *Apis mellifera* could detect 8% contrast at 0.09 cycles deg^−1^ (for square wave gratings). This is equivalent to a contrast sensitivity of at least 12.5 for the object discrimination system in this species, but the aim of this study was not to define the peak contrast sensitivity. Physiological measurements from honeybees viewing wide-field translational stimuli report contrast sensitivities that are twice as high (Bidwell and Goodman 1993). High contrast sensitivity for wide-field motion stimuli is also common in other insects. Physiological recordings of wide-field motion-sensitive neurons in flies, butterflies, hawk moths and hoverflies have measured contrast sensitivities between 20 and 100 (e.g., Dvorak et al. 1980; Maddess et al. 1991; O’Carroll et al. 1996; Stöckl et al. 2016; for review see O’Carroll and Wiederman 2014). This high contrast sensitivity for the motion detection system is believed to be due to the spatial integration of outputs of many motion detectors that each view different parts of the visual field (Dvorak et al. 1980; O’Carroll and Wiederman 2014) and is likely critical for controlling flight, as a lack of reliable visual input could lead to a loss of control or a collision with other objects or the ground.

At this point we can only speculate on why, in comparison to the visual system mediating flight control, *B. terrestris* has such a dramatically low contrast sensitivity in their object discrimination system (see discussion, Chakravarthi et al. 2016). One possible explanation is that the higher spatial constraint of the bees in this set-up limited their visual performance compared to the free flying bees in this study (Balamurali et al. 2015). Another possible explanation is that the increased visual field occupied by the patterns in the flight tunnel compared to the y-maze improves the contrast sensitivity of the visual system by stimulating significantly more photoreceptors. Further studies in other bee and insect species are needed to better understand this relationship.

## Electronic supplementary material

Below is the link to the electronic supplementary material.

Supplementary material 1 (DOCX 60 kb)
