# Supplementary material for: High contrast sensitivity for visually guided flight control in bumblebees
Source: J Comp Physiol A Neuroethol Sens Neural Behav Physiol. 2017 Sep 6;203(12):999–1006. doi: 10.1007/s00359-017-1212-6 (PMC5696488; doi:10.1007/s00359-017-1212-6)
Supplement: Supplementary file 1 — Supplementary material 1 (DOCX 60 kb) [file 359_2017_1212_MOESM1_ESM.docx]

**Supplementary tables.**

**Table S1: Median, Interquartile ranges average and standard deviation of lateral position and ground speed data for different spatial frequencies.**

| **Spatial frequency**  **(cycles deg^-1^)** | **Median of lateral position**  **(cm)** | **IQR1 (lateral position)**  **(cm)** | **IQR2 (lateral position)**  **(cm)** | **Average**  **lateral position**  **(cm)** | **Standard deviation**  **(cm)** | **Median of ground speed**  **(cm s^-1^)** | **IQR1 (ground speed)**  **(cm s^-1^)** | **IQR2 (ground speed)**  **(cm s^-1^)** | **Average ground speed**  **(cm s^-1^)** | **Standard deviation**  **(cm s^-1^)** |
| --- | --- | --- | --- | --- | --- | --- | --- | --- | --- | --- |
| 0.04 | 20.92 | 19.85 | 22.79 | 21.00 | 2.28 | 60.9 | 50.6 | 71.5 | 62.3 | 15.9 |
| 0.06 | 22.99 | 20.95 | 25.46 | 22.73 | 2.76 | 50.9 | 45.1 | 57.1 | 52.1 | 9.4 |
| 0.07 | 20.25 | 18.18 | 22.22 | 19.76 | 2.76 | 51.9 | 45.2 | 59.3 | 51.8 | 11.1 |
| 0.13 | 18.72 | 17.05 | 21.15 | 19.03 | 2.54 | 59.2 | 50.3 | 71.7 | 59.3 | 13.5 |
| 0.21 | 18.42 | 16.12 | 19.96 | 18.03 | 2.50 | 63.1 | 51.2 | 71.0 | 61.8 | 13.2 |
| 0.22 | 15.71 | 14.06 | 17.32 | 15.86 | 2.29 | 64.7 | 53.9 | 77.8 | 66.1 | 14.9 |
| 0.57 | 16.78 | 14.39 | 19.26 | 17.27 | 3.95 | 64.5 | 53.7 | 70.8 | 62.9 | 13.7 |
| Grey | 15.42 | 13.69 | 18.10 | 15.60 | 3.48 | 66.1 | 55.4 | 76.0 | 65.5 | 16.8 |

**Table S2: Median, Interquartile ranges average and standard deviation of lateral position for various contrasts for four low spatial frequency patterns.**

| **Spatial frequency**  **(cycles deg^-1^)** | **Michelson**  **Contrast**  **(%)** | **Median of lateral position**  **(cm)** | **IQR1 (lateral position)**  **(cm)** | **IQR2 (lateral position)**  **(cm)** | **Average**  **lateral position**  **(cm)** | **Standard deviation**  **(cm)** |
| --- | --- | --- | --- | --- | --- | --- |
| 0.04 | 0 | 15.42 | 13.70 | 18.01 | 15.60 | 3.48 |
|  | 3 | 21.36 | 19.09 | 23.90 | 21.09 | 3.78 |
|  | 14 | 22.47 | 20.40 | 24.23 | 22.30 | 2.34 |
|  | 22 | 22.58 | 20.95 | 23.82 | 22.18 | 2.64 |
|  | 39 | 22.00 | 19.72 | 24.29 | 21.88 | 2.59 |
|  | 87 | 20.92 | 19.85 | 22.79 | 21.00 | 2.28 |
| 0.06 | 0 | 15.42 | 13.70 | 18.01 | 15.60 | 3.48 |
|  | 3 | 22.41 | 20.47 | 24.01 | 22.29 | 2.73 |
|  | 14 | 21.82 | 19.62 | 23.12 | 20.50 | 3.58 |
|  | 22 | 22.48 | 20.45 | 23.43 | 22.13 | 2.04 |
|  | 39 | 21.65 | 19.04 | 22.87 | 20.60 | 2.86 |
|  | 87 | 22.99 | 20.95 | 25.46 | 22.73 | 2.76 |
| 0.07 | 0 | 15.42 | 13.70 | 18.01 | 15.60 | 3.48 |
|  | 3 | 22.43 | 20.56 | 23.89 | 22.31 | 2.28 |
|  | 14 | 20.75 | 19.77 | 23.68 | 21.42 | 2.58 |
|  | 22 | 22.20 | 20.81 | 23.87 | 22.01 | 2.38 |
|  | 39 | 19.83 | 18.19 | 21.36 | 19.42 | 2.78 |
|  | 87 | 20.25 | 18.18 | 22.22 | 19.76 | 2.76 |
| 0.13 | 0 | 15.42 | 13.70 | 18.01 | 15.60 | 3.48 |
|  | 3 | 20.07 | 18.92 | 21.94 | 20.44 | 2.20 |
|  | 14 | 19.72 | 18.37 | 22.35 | 20.02 | 2.85 |
|  | 22 | 20.94 | 17.60 | 22.80 | 20.27 | 2.99 |
|  | 39 | 18.72 | 15.29 | 20.78 | 18.23 | 3.47 |
|  | 87 | 18.72 | 17.05 | 21.15 | 19.03 | 2.54 |
